# Improvement of Soybean *Agrobacterium*-Mediated Transformation Efficiency by Adding Glutamine and Asparagine into the Culture Media

**DOI:** 10.3390/ijms19103039

**Published:** 2018-10-05

**Authors:** Li Chen, Yupeng Cai, Xiujie Liu, Weiwei Yao, Chen Guo, Shi Sun, Cunxiang Wu, Bingjun Jiang, Tianfu Han, Wensheng Hou

**Affiliations:** 1 National Center for Transgenic Research in Plants, Institute of Crop Sciences, Chinese Academy of Agricultural Sciences, Beijing 100081, China; chenli01@caas.cn (L.C.); caiyupeng2015@126.com (Y.C.); liuxiujie235@163.com (X.L.); yaoweiwei_nky@126.com (W.Y.); goldsion@163.com (C.G.); 2 Ministry of Agriculture Key Laboratory of Soybean Biology (Beijing), Institute of Crop Sciences, Chinese Academy of Agricultural Sciences, Beijing 100081, China; sunshi@caas.cn (S.S.); wucunxiang@caas.cn (C.W.); jiangbingjun@caas.cn (B.J.); hantianfu@caas.cn (T.H.)

**Keywords:** soybean, *Agrobacterium*-mediated transformation, l-asparagine, l-glutamine, transformation frequency

## Abstract

As a genetically modified crop, transgenic soybean occupies the largest global scale with its food, nutritional, industrial, and pharmaceutical uses.Efficient transformation is a key factor for the improvement of genetically modified soybean. At present, the *Agrobacterium*-mediated method is primarily used for soybean transformation, but the efficiency of this method is still relatively low (below 5%) compared with rice (above 90%). In this study, we examined the influence of l-glutamine and/or l-asparagine on *Agrobacterium*-mediated transformation in soybean and explored the probable role in the process of *Agrobacterium*-mediated transformation. The results showed that when the amino acids l-glutamine and l-asparagine were added separately or together to the culture medium, the shoot induction frequency, elongation rate, and transformation frequency were improved. The combined effects of l-glutamine and l-asparagine were better than those of l-glutamine and l-asparagine alone. The 50 mg/L l-glutamine and 50 mg/L l-asparagine together can enhance the transformation frequency of soybean by attenuating the expression level of *GmPRs* (*GmPR1*, *GmPR4*, *GmPR5,* and *GmPR10*) and suppression of the plant defense response. The transgene was successfully transmitted to the T1 generation. This study will be useful in genetic engineering of soybean.

## 1. Introduction

Soybean (*Glycine max* (L.) Merrill) is one of the world’s most important oil and protein crops, serving as a food source for humans and for animal forage, and it has industrial uses [1]. Transgenic soybean is a genetically modified (GM) crop that occupies the largest global scale with its food, nutritional, industrial, and pharmaceutical uses. From 1996 to 2015, approximately 1030 million hectares of GM soybean were planted and more than 50 billion US dollars were generated as income to farmers. In 2015, approximately 92.1 million hectares of GM soybean were planted around the world in 11 countries, which accounted for 51% of all GM crop hectares and 83% of the soybean grown all over the world [2].

Valuable genes and efficient transformation are the key factors for the improvement of GM soybean. As rice varieties are becoming more popular model plants, research on soybeantransgenic lines and the improvement of soybean agronomic characteristics has fallen behind, and that may be possibly due to genome complexity and the lower transformation efficiency [3].

A new technology for genome editing—the CRISPR (clustered regularly interspaced short palindromic repeat)/Cas (CRISPR-associated) system—has been successfully used for genome engineering in many important crops in recent years [4,5,6,7,8,9,10,11,12]. Since 2015, CRISPR/Cas9-mediated genome editing in soybean has shown an initial success [13,14,15,16,17,18,19]. This technology provides a powerful tool for accurate genetic modification and gene function identification, but it also relies on transformation efficiency.

Soybean transformation was first reported in 1988 using an *Agrobacterium tumefaciens* infection with cotyledonary node plant regeneration [20] or by particle bombardment of the meristems of immature seeds [21]. Over more than 20 years, these two methods have been continually improved and developed, and *Agrobacterium*-mediated transformation remains the method of choice in soybean.

Many factors are known to affect the efficiency of T-DNA delivery to the plant cell. The factors influencing *Agrobacterium*-mediated transformation efficiency include the *Agrobacterium* strain, *Agrobacterium* inoculation density, explant types, genome, inoculation period, culture medium, and selection system, among others [22,23]. According to these factors, much effort has been made to enhance the efficiency of soybean transformation: the utilization of different explants, such as the cotyledonary node, hypocotyls, embryonic tip, half-seed, and immature seed [24,25,26,27]; different selection agents, such as glufosinate, glyphosate, and hygromycin [28,29,30]; supplementation with amino acids or antioxidants, such as dithiothreitol, l-cysteine, and sodium thiosulfate [30,31], among others [32,33]. 

It has been reported that the addition of l-glutamine into culture medium alone or in combination with a cold shock pretreatment could enhance *Agrobacterium* transformation efficiency [34]. l-Glutamine was also found to inhibit β-aminobutyric acid (BABA)-induced plant resistance to a bacterial pathogen of *Arabidopsis* [35]. The metabolism of glutamine has been implicated in the plant response to pathogen infection [36]. The molecular mechanisms by which glutamine affects plant defence responses are still poorly understood, but the results suggest that l-glutamine could play a role in lessening host defence responses by attenuating the expression of certain pathogenesis-related genes (*PRs*) and potentially improve the efficiency of *Agrobacterium*-mediated plant transformation [34,37].

In the present study, we examined the influence of l-glutamine and/or l-asparagine on *Agrobacterium*-mediated transformation in soybean and explored the probable role of l-glutamine and/or l-asparagine during the process of *Agrobacterium*-mediated transformation. We designed experiments to: determine the effects of l-glutamine and/or l-asparagine, which were added to the co-cultivation medium (CCM), recovery medium (SIM0), selection medium (SIM6), shoot elongation medium (SEM), and rooting culture medium (RCM),on shoot regeneration and elongation; to investigate if these modified media could attenuate plant defense response, enhance the transformation frequency, and further contribute to a wider range of soybean genotypes, and also; to investigate the transmission of the transgene to the T1 generation. 

## 2. Results

### 2.1. l-Asparagine and l-Glutamine Improved the Shoot Induction Frequency and Increased the Frequency of Highly-Expressing Transgene (GUS) Shoots

The cultivar Jack, routinely used in soybean transformation for its relatively high transformation frequency between 3.5–5.5%, was selected to test the treatments. After co-cultivation and recovery, the explants were placed on selection medium, and induced multiple shoots at the growing point of the cotyledon and hypocotyls. The shoot induction frequency was approximately 68.9% when no l-asparagine or l-glutamine was added to the media, and the shoot induction frequencies were approximately 77.9% and 84.3% in media supplemented with l-asparagine or l-glutamine, respectively. The frequency was enhanced by 10–15% in media supplemented with l-asparagine or l-glutamine. The shoot induction frequency was above 90% when media was supplemented with both l-asparagine and l-glutamine. Compared with no l-asparagine or l-glutamine was added to the media, the frequency was enhanced remarkably when media was supplemented with both l-asparagine and l-glutamine (Table 1).

**Figure 1:**
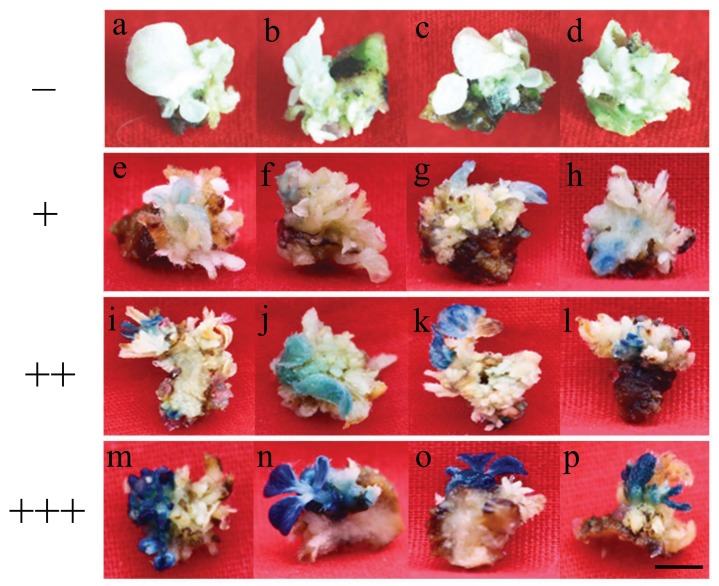
The fourclasses of multiple shoots for transgene (GUS) staining. (**a**–**d**)“−“Representation of negative; (**e**–**h**)“+” representation of low dyeing; (**i**–**l**)“++” representation of medium dyeing; (**m**–**p**)“+++” representation of high dyeing. Scale bar, 1 cm.

All evaluations in transformed soybean plants were conducted using the expression cassette which encodes for a GUS reporter gene and Bar resistance gene for selection. The reporter transgene expression (GUS) of multiple shoots was tested and classified according to staining intensity as measured by ELISA. According to the GUS activity, the GUS staining was classified into four classes: negative (−) (Figure 1a–d), the GUS activity was below 0.45 U/g; low (+)(Figure 1e–h), the GUS activity was around 0.45 to 0.6 U/g; medium (++)(Figure 1i–l), the GUS activity was around 0.6 to 0.7 U/g; and high (+++) (Figure 1m–p), the GUS activity was around 0.7 to 0.8 U/g. For each treatment, 30 shoots were evaluated for GUS staining as classified in Figure 1 and GUS activity as measured by ELISA.

As shown in Table 2, in the presence of l-aspargine the percentage of GUS activity was 66.7%, while in the presence of l-glutamine activity rose to 70%, in comparison to shoots in the absence of l-amino acids tested. In the presence of l-asparagine and l-glutamine overall levels of GUS-positive shoots was 70%. It suggested that l-asparagine and l-glutamine could increase the frequency of highly-expressing GUS shoots.

### 2.2. l-Asparagine and l-Glutamine Improved the Elongation Efficiency and Prolonged Transfer for Shoot Elongation (SE)

After selecting for shoot induction, the explants produced multiple herbicide-resistant buds. The multiple buds without cotyledons were transferred to the shoot elongation medium (SEM) and subcultured for 3–5 times. Therefore, we recorded the number of elongated plants cultured every time (Table 3). When the media with no l-asparagine or l-glutamine added, 28.4% of the elongated shoots were produced in the first time (SE1); 37.8% and 33.8% of the elongated shoots were produced in the second and third times (SE2 and SE3), respectively. There were no elongated shoots in the fourth and fifth times (SE4 and SE5). When the media was supplemented with l-asparagine, 34.6% of the elongated shoots were produced in SE1; 43.8% and 19.1% of the elongated shoots were produced in SE2 and SE3, respectively. Only 2.5% of the elongated shoots were produced in SE4. When the media was supplemented with l-glutamine, 13.8%, 37.1%, 34.5%, 11.2%, and 3.4% of the elongated shoots were produced in SE1–SE5, respectively. When both l-asparagine and l-glutamine were added to the media, 13.3%, 57.7%, 19.5%, and 9.5% of the elongated shoots were produced in SE1–SE4, respectively.

At this stage, after the length of the shoots had elongated to more than 4 cm, the shoots were cut from the multiple buds and then transferred for rooting. The more elongated shoots available, the more transgenic plants can be cultured. Table 3 shows the total number of elongated plants and the elongation rate. When the media with no l-asparagine or l-glutamine added, the elongation rate was approximately 20.2%. When the media was supplemented with l-asparagine or l-glutamine, the elongation rate was approximately 39.8% and 26.6%, respectively. When both l-asparagine and l-glutamine were added to the media, it was evidently enhanced and reached to 41.6%. It suggested that when supplemented with either l-asparagine, l-glutamine or in combination, the elongation rates were significantly higher than in the absence of the l-amino acids.

### 2.3. l-Asparagine and l-Glutamine Improved the Transformation Frequency

**Figure 2:**
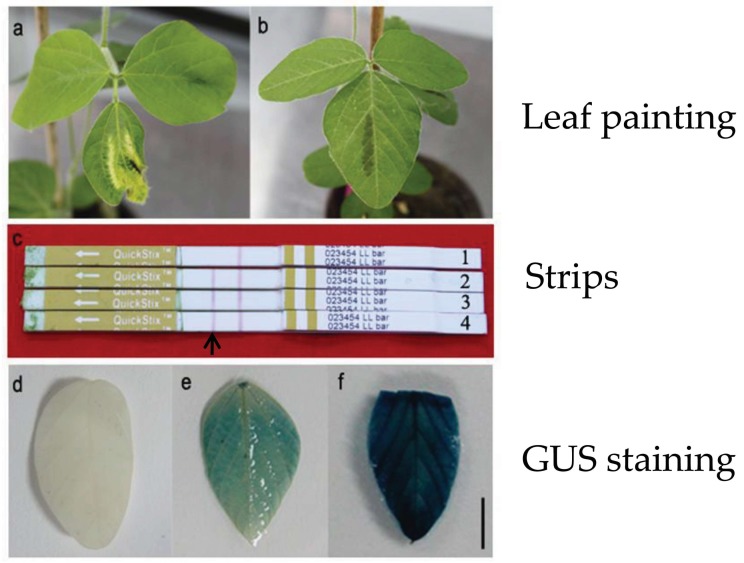
Detection of transgenic plants. (**a**) Negative plants by leaf painting; (**b**) Positive plants by leaf painting; (**c**) Strips detection: 1 Negative plant, 2–4 Positive plants, the bands at black arrowhead indicate that bar is positive; (**d**) Negative plants by GUS staining;(**e**,**f**) Positive plants by GUS staining. Scale bar, 1 cm.

We cut leaves from the elongated shoots to detect the positive shoots, and verification was performed using GUS staining, PAT strips and leaf painting (Figure 2). Strips were used to determine the presence of PAT protein of transgenic plants, and leaf painting was used to check the resistance of *bar* gene. When the media with no l-asparagine or l-glutamine added, 74 elongated shoots were detected, and among them, 12 shoots tested positive by PAT strips. The positive rate of the elongated shoots was approximately 16.2%. The positive shoots were 14.8% and 36.0% in media supplemented with l-asparagine or l-glutamine, respectively. 46 of 180 elongated shoots were tested positive, and the positive rate of the elongated shoots was approximately 30.2% in when media was supplemented with both l-asparagine and l-glutamine (Table 4). The leaf painting and GUS staining were performed in order to corroborate results between the two methods and there was no difference between methods, so only PAT results are presented in Table 4.

From the detection of the elongated shoots, we calculated the transformation frequency. As shown in Table 4, the transformation frequency was approximately 3.5% with no l-asparagine or l-glutamine was added to the media. The transformation frequency values were approximately 5.9% and 8.8% in media supplemented with l-asparagine or l-glutamine, respectively. The transformation frequency reached 11.1% in when media was supplemented with both l-asparagine and l-glutamine. The results indicated that l-asparagine and l-glutamine could remarkably enhanced transformation frequency.

### 2.4. The Transformation Frequency in Four Soybean Cultivars Utilizing the New Conditions

According to the results of the different treatments, the transformation frequency was the highest when media was supplemented with both l-asparagine and l-glutamine for cultivar Jack. Four soybean cultivars Jack, Williams 82 (standard cultivar), Zigongdongdou (early-maturity cultivar) and Heihe 27 (late-maturity cultivar) were infected with *Agrobacterium* and cultured on the improved medium supplemented with both l-asparagine and l-glutamine. The media with no l-asparagine or l-glutamine was the control. Shoots were regenerated with 6 mg/L glufosinate for all four cultivars. Under the new media supplemented with both l-asparagine and l-glutamine, the shoot induction frequency in Jack reached 92.3%, and in Williams 82 and Heihe 27, it was above 85%. The shoot induction frequency was approximately 62.7% in Zigongdongdou. Compared with the media with no l-asparagine or l-glutamine, the shoot induction frequency was enhanced in all cultivars tested. In addition, the transformation frequency was also improved. The transformation frequency in Jack was the highest. In Williams 82 and Heihe27, the transformation frequency was above 8%. It was lower in Zigongdongdou (Table 5). The results demonstrated that the improved conditions can be applied to more soybean cultivars.

### 2.5. Analysis of T1 Plants

**Figure 3:**
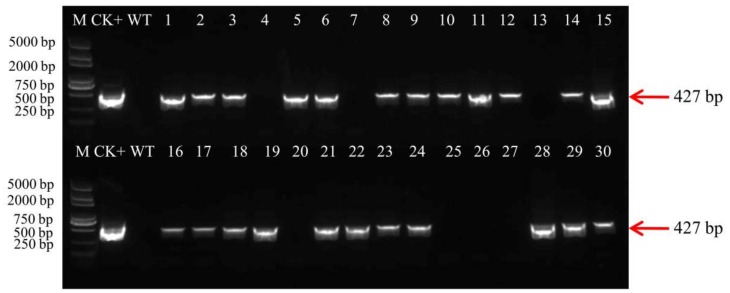
Detection of T1 transgenic plants by PCR. PCR analysis of genomic DNA of T1 transgenic plants using *bar* gene primers. The length of PCR product is 427 bp. M: DL2000 Plus; CK+: plasmid DNA; WT: wild-type soybean plant; lanes 1–7: T1 lines (line 1-1, line 1-2, line 2-1, line 3-1, line 3-3, line 3-4, line 4-1) versus 1–4 T0 lines from treatment without l-amino acid;lanes 8–15: T1 lines (line 6-1, line 6-2, line 7-1, line 7-2, line 7-3, line 7-4, line 7-5, line 8-1) versus 6–8 T0 lines from treatment with l-asparagine;lanes 16–20: T1 lines (line 11-1, line 12-3, line 13-2, line 14-5, line 15-1)versus 11–15 T0 lines from treatment with l-glutamine;lanes 21–30: T1 lines(line 16-1, line 17-1, line 17-2, line 17-9, line 17-10, line 17-11, line 17-12, line 17-14, line 18-5, line 19-10) versus 16–19 T0 lines from treatment with both l-asparagine and l-glutamine).

**Figure 4:**
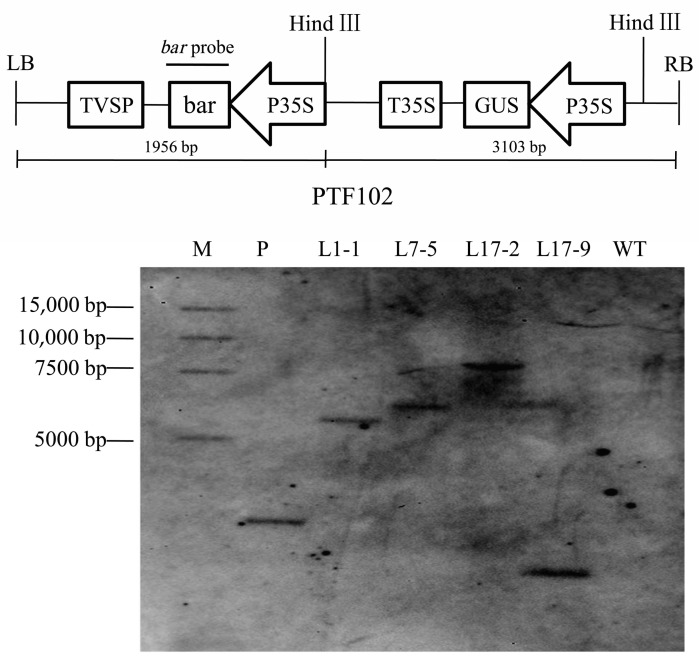
Southern blot analysis of T1 transgenic plants. Plasmid and genomic DNA were digested with HindIII, and hybridized with the bar probe labeled with DIG. M: marker; P: plasmid DNA of pWMB123; L1-1: DNA from T1 plant with bar^+^ from transgenic line 1; L7-5: DNA from T1 plant with bar^+^ from transgenic line 7; L17-2 and L17-9: DNA from T1 plant with bar^+^ from transgenic line 17. WT: DNA from nontransgenic plants.

Five independent T0 transformants of each treatment were randomly selected, and the T1 plants from the five independent T0 transformants were examined for the presence of the *bar* gene by PCR. Some PCR detection results were showed in Figure 3. The plants were positive by presence of the amplified fragment of 427 bp, and the negative plants were not amplified in any fragment (Figure 3). The T1 plants were also examined the segregation ratios by Chi-square analysis (Table 6). The results indicated that only three lines (line 1, line 7, line 17) showed a 3:1 ratio (*bar^+^*:*bar^–^*), and other lines were not fit to this ratio. We further confirmed the copy number of T1 transgenic plants (line 1-1, line 7-5, line 17-2, line 17-9) using Southern blot. The results showed the T1 plants in these three T0 lines had only one copy of the *bar* gene (Figure 4).

Next, the seeds collected from all the other T0 transformants were planted and tested. If only one of the T1 plants was detected positive which was confirmed both by PCR and strip, we considered the T0 transformant was inherited to T1 generation. We determined that 66.7% (8 out of 12) in treatment with no l-asparagine or l-glutamine, 68.2% (15 out of 22) in treatment with only l-asparagine, 71.9% (23 out of 32) in treatment with only l-glutamine, and 73.9% (34 out of 46) in treatment with both l-asparagine and l-glutamine *bar* positive T0 transformants had inherited in T1 plants, respectively (Table 7). Some of the T0 transformants in different treatments did not produce positive plants in T1 plants. Transgene positive T0 soybean plants did not necessarily pass the transgene to the next generation. There was no obvious difference in inheritance frequency between treatments. This indicated that l-asparagine and l-glutamine could not improve chimera.

### 2.6. l-Asparagine and l-Glutamine Reduce Plant Defense Response by Regulating Pathogen-Related Genes

**Figure 5:**
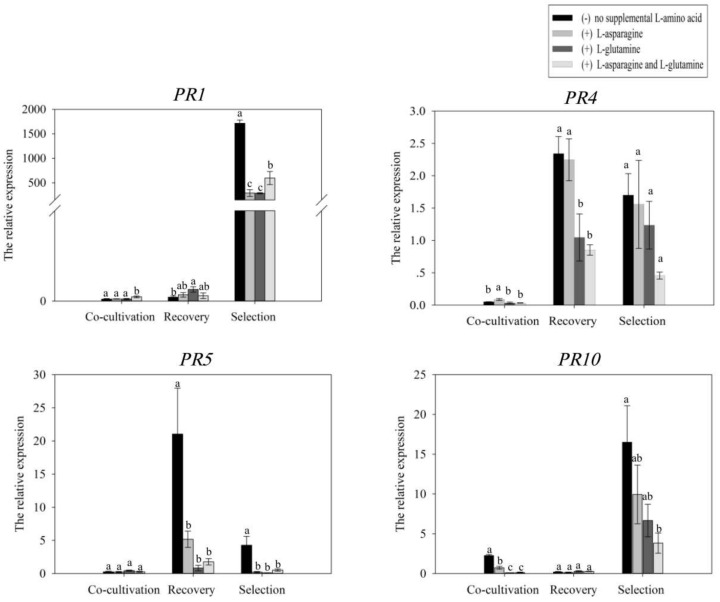
The expression level of *GmPRs*in different culture stage with treatments by qRT-PCR. The expression level of *GmPR1*, *GmPR4*, *GmPR5*, and *GmPR10* were analyzed during co-cultivation, recovery and selection culture.*GmActin* was used as an internal reference. The different small letters represent significant differences between treatments by ANOVA (*p* < 0.05).

Some soybean *PRs* genes were reported that they were induced by wounding and wall glucan elicitor treatment which is a pathogen-derived general defense elicitor [38,39]. To evaluate whether the plant defense response was altered with different treatments, the expression of soybean *PRs*, *GmPR1*, *GmPR4*, *GmPR5* and *GmPR10* were analyzed during co-cultivation, recovery and selection culture. The expression of these *GmPRs* in response to the treatments during different culture stage was different. The results showed that the expression of *GmPR1* was weak with different treatments during co-cultivation and recovery culture, and strongly induced during selection culture in all treatments. The level of *GmPR1* in treatment with no l-asparagine or l-glutamine was higher than that in other treatments with l-asparagine or/and l-glutamine. The expression level of *GmPR4* was induced strongly in recovery and selection culture. The level of *GmPR4* in treatment with no l-asparagine or l-glutamine was highest, and in treatment with both l-asparagine and l-glutamine it was lowest. The expression of *GmPR5* has no obvious difference among treatments in co-cultivation culture, and it was obviously different between the treatment with no l-asparagine or l-glutamine and other three treatments in recovery and selection culture. The expression level of *GmPR10* was the highest in treatment with no l-asparagine or l-glutamine in co-cultivation and selection culture. There was no obvious difference among treatments in recovery culture (Figure 5). The remarkable difference in expression of these *GmPRs* in various treatments suggested that l-asparagine and l-glutamine could play a role in mitigating plant defense responses by attenuating the expression level of *GmPRs*. 

## 3. Discussion

*Agrobacterium*-mediated soybean transformations have been previously reported, but the transformation frequency has remained low. The effects of the great complexity of soybean genotypes and different explants selections on soybean transformation have been studied by different laboratories [24,25,26,27,31,32]; however, none of the reported protocols appear to be sufficient.

l-Asparagine and l-glutamine are the two major nitrogen transport compounds in most plants [40]. Under normal growth conditions, the C/N ratio in the plant is probably the most important determining factor for whether asparagine or glutamine is selected as the major nitrogen. A high C/N ratio can lead to the production of glutamine for nitrogen transport, and a low C/N ratio lead to the production of asparagine for nitrogen transport [41,42]. In addition to providing carbon and nitrogen, glutamine is also involved in other cellular processes, including antioxidative stress, mTOR signalling, autophagy, and plant defence reactions [34]. The *Agrobacterium*-mediated transformation efficiency in perennial ryegrass (*Lolium perenne* L.) was highly improved by *myo*-inositol removal, cold shock pretreatment and l-glutamine supplementation, which enhanced *Agrobacterium* binding to the cell surface and decreased H_2_O_2_ production [34]. As the pathogen competes for host nitrogen reserves during infection, the depletion of l-glutamine in infected tissues could activate plant defence responses. In addition, a direct application of glutamine on leaves can strongly inhibit H_2_O_2_ production and could suppress cell death during pathogen infection [43].

In some labs, l-asparagine and l-glutamine have been added into the culture medium of at certain stages, like in shoot elongation medium or rooting medium [24,44]. However, in our study, we added the l-asparagine and/or l-glutamine from co-culture to rooting, the results showed that l-asparagine or l-glutamine added alone to culture media could enhance the regeneration rate, but better results were seen with the combination of l-asparagine and l-glutamine (Table 1). When media was supplemented with both l-asparagine and l-glutamine, the shoot induction efficiency was enhanced to 90%. The elongation rate was also increased when the medium had both l-asparagine and l-glutamine; the elongation rate was above 40%. For soybean, not all multiple shoots could elongate, only the elongated shoots can further grow into transformant plant. A high elongation rate is the basis for obtaining a high transformation frequency. 

For the typical Jack cultivar, utilizing l-asparagine and l-glutamine in the culture medium increased the soybean transformation efficiency to approximately 11%, an increase from approximately 3.5–5.5% when no asparagine or glutamine was added to the media (Table 4 and Table 5). In addition, this increase in overall transformation efficiency was applicable to other soybean cultivars, with the exception of Zigongdongdou (Table 5) which for reasons unknown is relatively recalcitrant to *Agrobacterium* transformation. Based on this improved transformation, the soybean genome editing (CRISPR/Cas9) was successfully achieved in our lab [45]. Although the improved transformation efficiency was still not high, we can see that it has the potential to enhance the transformation efficiency by improving the positive rate of elongated shoots. At present, through this transformation process, high shoot induction and elongation rates can be achieved, and elongated shoots can be produced, although many were not positive. Further optimization of glufosinate selection may be the key factor to improving the soybean transformation efficiency.

The increased transformation efficiencies resulted in an overall larger number of T0 and T1 plants under new conditions tested. The number of inheritance transformants was 8 for no supplement versus 34 for l-asaparagine and glutamine (Table 7). A larger number of genetically stable transformants can be screened by a piece of leaf for the presence of PAT (strip tested) and confirmed through either PCR or activity of the transgene (GUS) during early seedling development. Perhaps even growing T0 seeds on selection media could help screen transformants. So the T1 plants with the desired outcome can be quickly detected from the larger number of T1 plants.

Plant defense responses can lead to cell death at the sites of *Agrobacterium* infection and lower transformation frequencies. Our results showed that the expression of *GmPRs* in various treatments was remarkable difference during co-cultivation, recovery and selection culture. The expression level of *GmPRs* among treatments was relative low during co-cultivation culture. The expression level of *GmPR4* and *GmPR5* was induced during recovery culture, but it was higher in treatment with no l-asparagine or l-glutamine than in other treatments. During selection culture, the expression level of *GmPR1*, *GmPR4*, *GmPR5* and *GmPR10* was higher in treatment with no l-asparagine or l-glutamine than in other treatments. These results suggested that l-asparagine and l-glutamine could play a role in mitigating plant defense responses by attenuating the expression level of *GmPRs*. The transformation frequency was enhanced in treatments with l-asparagine or/and l-glutamine. And this enhancement in transformation frequency was likely attributable to attenuation in the expression of *GmPRs* and suppression of plant defense response by l-asparagine and l-glutamine.

## 4. Materials and Methods

### 4.1. Plant Materials

**Figure 6:**
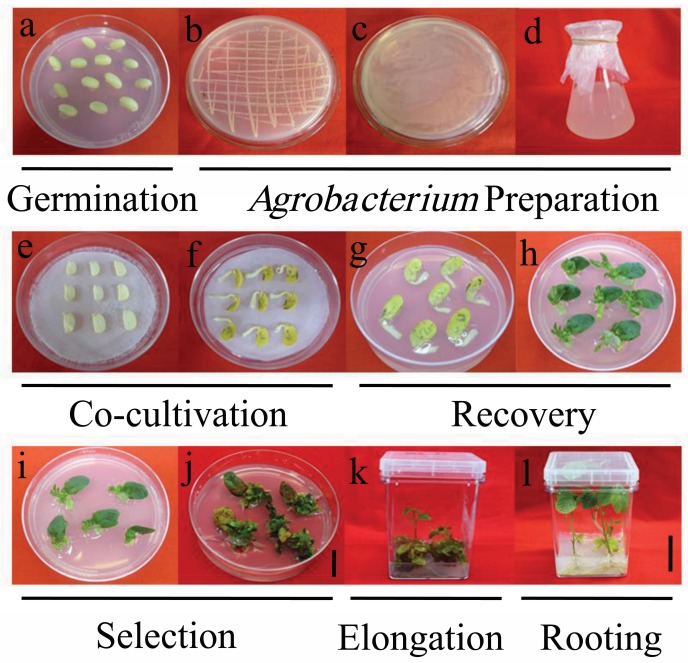
The process of *Agrobacterium*-mediated soybean half-seed transformation. (**a**) Germination; (**b**–**d**)*Agrobacterium* preparation; (**e**) Co-cultivation 0 day; (**f**) Co-cultivation 5 days; (**g**) Recovery 0 day; (**h**) Recovery 7 days; (**i**) Selection 0 day; (**j**) Selection 21 days; (**k**)Elongation; (**l**) Rooting 7 days. Scale bars, (**a**–**j**) 2.5 cm; (**k**,**l**) 4 cm.

The soybean cultivars (Jack, Zigongdongdou, Williams 82 and Heihe 27) were utilized for *Agrobacterium*-mediated transformation in the present study, and Jack was used for the culture medium experiments. Healthy seeds were surface-sterilized by exposure to chlorine gas for 16 h as described by Di [46]. Sterilized seeds were placed in germination culture medium (GCM) containing 3.1 g/L Gamborgs Basal Salt Mixture (Phytotech, G768, Lenexa, KS, USA), 20 g/L sugar, 1mL/LGamborgs Vitamin Solution (Phytotech, G219, Lenexa, KS, USA) and 7 g/L agar (Sigma, St. Louis, MO, USA), pH 5.8, and the seeds were germinated at 25 °C for 18–20 h on the light (Figure 6a).

### 4.2. Agrobacterium Strain and Vector

*A. tumefaciens* strain EHA101 containing the binary vector PTF102 was used in the experiments. The vector carried T-DNA containing the β-glucuronidase gene (*uidA*) driven by the 35S CaMV promoter and the *bar* gene as an herbicide resistance marker [44,47].

### 4.3. Agrobacterium Preparation

*Agrobacterium* strain stocks of EHA101/PTF102 stored at −80 °C were streaked on solidified YEP medium plates containing 5 g/L NaCl, 10 g/L tryptone, 5 g/L yeast extract, and 15 g/L agar, with 50 mg/L kanamycin, 75 mg/L chloromycetin, 75 mg/L spectinomycin, and 50 mg/L rifampicin. Plates streaked with *Agrobacterium* were incubated at 28 °C for approximately 2 days until colony formation (Figure 6b). The colonies were collected by a spreader, daubed onto new solidified YEP medium plates with the same antibiotics and incubated overnight at 28 °C (Figure 6c). The fresh *Agrobacterium* were resuspended in liquid co-cultivation medium (LCCM) containing 1/2 Murashige&Skoog Basal Salt Mixture (Phytotech, M524, Lenexa, KS, USA), 3.9 g/L2-(*N*-Morpholino) ethanesulfonic acid(MES), 30 g/L sugar, 1mL/L Gamborgs Vitamin Solution, 150 mg/L dl-Dithiothreitol (DTT), 2 mg/L zeatin and 40 mg/L 3′,5′-dimethoxy-4-hydroxyacetophenone(AS), pH 5.4.Next, a final optical density of 0.6 was measured at 600 nm, and the *Agrobacterium* cultures were prepared for transformation (Figure 6d).

### 4.4. Infection and Co-Cultivation

Explants were prepared from one-day-old seedlings following the method described by Paz [24]. A longitudinal cut along the hilum was made to separate the cotyledons, and the seed coat was removed. The embryonic axis found at the junctions of the hypocotyls and the cotyledon was excised to obtain the half-seed explants. The explant cuttings were immersed in *Agrobacterium* for 2 h at 50 rpm. After inoculation, each of the 9 cotyledons were placed in solid co-culture medium (CCM) containing1/2 Murashige&Skoog Basal Salt Mixture, 3.9 g/L MES, 30 g/L sugar, 1mL/L Gamborgs Vitamin Solution, 150 mg/L DTT, 40 mg/L AS, 2 mg/L zeatinand 7 g/L agar, pH 5.4, with a piece of Whatman filter paper and then incubated at 22 °C in the dark for 5 days (Figure 6e,f).

### 4.5. Recovery Culture and Selection Culture

After co-cultivation, explants were then transferred to recovery medium (SIM0) containing 3.1 g/L Gamborgs Basal Salt Mixture, 0.98 g/L MES, 30 g/L sucrose, 1 mL/L Gamborgs Vitamin Solution, 150 mg/L cefotaxime, 450 mg/L timentin, 1 mg/L 6-Benzylaminopurine (6-BA), and 7 g/L agar, pH 5.7, and incubated at 28 °C for 7 days (Figure 6g,h). Seven days after recovery, the explants were transferred to selection culture medium (SIM6) containing 3.1 g/L Gamborgs Basal Salt Mixture, 0.98 g/L MES, 30 g/L sucrose, 1 mL/L Gamborgs Vitamin Solution, 150 mg/L cefotaxime, 450 mg/L timentin, 1 mg/L 6-BA, 7 g/L agar, and 6 mg/L glufosinate, pH 5.7, and incubated at 28 °C for 21 days (Figure 6i,j).

### 4.6. Shoot Elongation and Rooting

After selection culture, the cotyledons and brown leaves were cut from the explants, and the remaining tissues were transferred to shoot elongation medium (SEM) containing 4.0 g/L Murashige&Skoog Basal Salt Mixture, 0.6 g/L MES, 30 g/L sucrose, 1 mL/L Gamborgs Vitamin Solution, 150 mg/L cefotaxime, 450 mg/L timentin, 0.1 mg/L 3-Indoleacetic acid(IAA), 0.5 mg/L Gibberellic acid(GA), 1 mg/L zeatin, 7 g/L agar, and 6 mg/L glufosinate, pH 5.6, and incubated at 28 °C(Figure 6k). The culture medium was changed every two weeks. Simultaneous with changing the SEM, the elongated shoots (5–8 cm) were cut from the base of the buds, and the stems were dipped in 1 mg/L Indole-3-Butytric acid (IBA) for 1 min, placed ina rooting culture medium (RCM) containing ½ Murashige&Skoog Basal Salt Mixture, 0.6 g/L MES, 20 g/L sucrose, 1 mL/L Gamborgs Vitamin Solution, and 7 g/L agar,3 mg/L glufosinate, pH 5.7, and incubated at 28 °C for 7 days (Figure 6l). After root production, the plants were transferred topots and grown in the greenhouse.

### 4.7. Adding l-Asparagine and l-Glutamine

To determine the effects of l-asparagine and l-glutamine on *Agrobacterium*-mediated transformation efficiency, different experimental treatments were prepared. Starting with the co-cultivation medium, l-asparagine and/or l-glutamine was added to five different medium types (CCM, SIM0, SIM6, SEM, RCM): (1) with no l-asparagine or l-glutamine; (2) with 100 mg/L l-asparagine; (3) with 100 mg/L l-glutamine; (4) with 50 mg/L l-asparagine and 50 mg/L l-glutamine.

### 4.8. Detection of the Shoot Induction Rate and Elongation Rate

During the selection stage, the shoot induction rate was calculated as follows: shoot induction rate = explants with shoots/total explants for infection × 100%. During the elongation stage, the elongation rate was calculated as follows: elongation rate = total elongated shoots/total explants for infection × 100%.

### 4.9. Detection of Positive Plants and Transformation Frequency

The transgenic plants were verified by GUS staining, leaf painting, and strip analysis. The leaf was incubated in GUS staining buffer (50 mM sodium phosphate, pH7.0, 0.5 mM potassium ferrocyanide, 0.5 mM potassium ferricyanide, 0.5 mg/mL 5-bromo-4-chloro-3-indolyl-β-d-glucuronide (X-Gluc), 0.1% Triton X-100 and 20% methanol) for 24 h at 37 °C and then washed in 95% ethanol before being photographed [48]. Next, 160mg/L of glufosinate was brushed on the leaf for a leaf painting test, and Liberty Link strips were used to detect PAT proteins in the transgenic plants. The plants used to calculate transformation frequency were all positive as determined by these three detection tests. The transformation frequency was calculated as follows: transformation frequency = the number of positive plants/the total explants for infection × 100%.

### 4.10. Detection of GUS Activity

0.1 g tissue samples were homogenized with 1 mL Phosphate buffer saline (PBS) buffer (pH 7.4) at 8000 rpm and 4 °C for 30 min, and the 50 μL supernatant was used for the reaction. The GUS activity was measured using an enzyme-linked immunosorbent assay (ELISA) kit (SU-B91122) according to the kit instructions. The GUS ELISA Kit includes a set of calibration standards. The calibration standards are assayed at the same time as the samples and produce a standard curve of Optical Density versus GUS activity. The GUS activity (U/g) is determined by comparing the Optical Density of the samples to the standard curve generated from standard with kit.

### 4.11. Plant Defence Analysis by qRT-PCR 

The expression of *GmPRs* was used for analysis of plant defense by qRT-PCR. The 5 days co-cultivated explants, 7 days recovery explants and 21 days selected explants were harvested for RNA extraction. Total RNA was isolated from frozen tissue using TransZolUp Plus RNA Kit (TransGen Biotech, Beijing, China). For reverse transcription, 1 μg of total RNA was used to synthesize single-stranded cDNA using TransScript One-Step gDNA Removal and cDNA Synthesis SuperMix (TransGen Biotech). The *GmActin* gene was used as internal control. The *GmPR1*, *GmPR4*, *GmPR5* and *GmPR10* were used for analysis of plant defense. The primers used for *GmActin* amplification were 5′-GAGCTATGAATTGCCTGATGG-3′ (forward) and 5′-CGTTTCATGAATTCCAGTAGC-3′ (reverse). The primers used for *GmPR1* amplification were 5′-ACACAGGTCGTTTGGGCTAA-3′ (forward) and 5′-CAACAAAGTTGCCAGGGGGA-3′ (reverse). The primers used for *GmPR4* amplification were 5′-TGAACGCCGTGAGTGCTTAT-3′ (forward) and 5′-CTGTATTCGTCACCCGGAGG-3′ (reverse). The primers used for *GmPR5* amplification were 5′-GTGCTTGGCGTTGAATACGG-3′ (forward) and 5′-TGTGGGACACGCATTCTTGA-3′ (reverse). The primers used for *GmPR10* amplification were 5′-ACAACGTCATCCCAAAGGCT-3′ (forward) and 5′-TCCATTCATTAACATCAGCCTCA-3′ (reverse). qRT-PCR reactions were performed according to the three-step method using SYBR Green I dye and the ABI7500 instrument for qRT-PCR. For qRT-PCR, a total volume of 20 μL was used that contained 10 μL of SYBR Premix Ex Taq (2×), 0.4 μL of dye, 0.4 μL of 10 μM of the upstream or downstream primers, 2 μL of cDNA template, and 6.8 μL of ddH_2_O. qRT-PCR amplification using the standard three-step procedure for denaturation was performed as follows: 95 °C for 30 s, followed by 40 cycles of 95 °C for 5 s, and 60 °C for 30 s. The relative expression level was calculated using the comparative 2^−ΔΔ*C*t^ method.

### 4.12. Molecular Analysis

PCR, strip and southern blot were used to analysis T1 generations of transgenic lines. Total genomic DNA was extracted from the leaves of the T1 transgenic plants using CTAB method. The *bar* gene detection in transgenic plants was demonstrated by PCR amplification of a 427 bp fragment using the primer pair 5′-GCACCATCGTCAACCACTACATC-3′ and 5′-CAGAAACCCACGTCATGCCAGTT-3′. For southern blot analysis, the 10 μg genomic DNA was digested with HindIII, the restriction products were separated on 0.8% agarose gel and transferred onto a nylon Hybond-N^+^ membrane (Roche, Mannheim, Germany) with a membrane transfer instrument (Model 785, Bio-Rad, Hercules, CA, USA). The *bar* (427 bp) PCR products were labelled with Digoxigenin and used as probes to hybridize with the digested DNA on the membrane. The hybridization and detection steps were performed according to the instructions for the DIG High Prime DNA Labeling and Detection Starter KitII (Roche, Mannheim, Germany).

### 4.13. Statistical Analysis

For all experiments, each treatment contained three replicates. The regeneration frequency, elongation rate and transformation frequency were expressed as the mean ± standard deviation, and the data shown represent the mean of three independent experiments. The data were analysed by ANOVA. The different small letters represent significant differences at *p*<0.05.

## 5. Conclusions

Adding 50 mg/L l-asparagine and 50 mg/L l-glutamine in culture medium enhanced shoot induction frequency and elongation rate in soybean transformation and finally improved the transformation frequency. The transformation frequency of Jack increased from a baseline of approximately 3.5–5.5% to 11% in the presence of both l-aparagine and l-glutamine. In addition, the improved conditions can be applied to more soybean cultivars. l-asparagine and l-glutamine could play a role in mitigating plant defense responses by attenuating the expression level of *GmPRs*. The enhancement in transformation frequency was likely attributable to attenuation in the expression of *GmPRs* and suppression of plant defense response. The transgene can be successfully transmitted to the T1 generation. This study may be useful in genetic engineering of soybean.

## Figures and Tables

**Table 1:** Shoot induction frequenciesofthe different treatments.

Treatment	No. of Explants	No. of Explants with Shoots	Shoot Induction Frequency (%)
(−) no supplemental l-amino acid	390	270	68.9 ± 2.0 ^d^
(+) l-asparagine	407	316	77.9 ± 2.0 ^c^
(+) l-glutamine	358	304	84.3 ± 1.3 ^b^
(+) l-asparagine and l-glutamine	431	400	92.5 ± 2.6 ^a^

Three independent experiments were performed for each treatment. No. of explants: the total number of explants for the three experiments; No. of explants with shoots: the total number of explants produced shoots for the three experiments. The explants with shoots were defined as the shoots with green leaves after 21 days transferred to selection media. The shoot induction frequency is expressed as the mean ± standard deviation. The different small letters represent significant differences between treatments by ANOVA (*p* < 0.05). (–) represents no L-amino acid added into the media. (+) represents L-amino acid added into the media.

**Table 2:** Expression ofmultiple shoots as determined by GUS staining and GUS activity.

Treatment	No. of Shoots in the Different Degrees of Gus Staining (Percentage, %)					No. of Total GUS-Positive Shoots ^a^ (Percentage, %)
	GUS StainingGUS Activity (U/g)	−(0–0.45)	+(0.45–0.6)	++(0.6–0.7)	+++(0.7–0.8)	
(−) no supplemental l-amino acid		18 (60.0)	5 (16.7)	5 (16.7)	2 (6.6)	12 (40.0)
(+) l-asparagine		10 (33.3)	8 (26.7)	6 (20.0)	6 (20.0)	20 (66.7)
(+) l-glutamine		9 (30.0)	11 (36.7)	7 (23.3)	3 (10.0)	21 (70.0)
(+) l-asparagine and l-glutamine		9 (30.0)	3 (10.0)	6 (20.0)	12 (40.0)	21 (70.0)

For each treatment, 30 shoots was for GUS staining. −: negative, GUS activity was below 0.45 U/g; +: low GUS staining, GUS activity was around 0.45 to 0.6 U/g; ++: medium GUS staining, GUS activity was around 0.6 to 0.7 U/g; +++: high GUS staining, GUS activity was around 0.7 to 0.8 U/g. 0.1 g tissues were used to measure GUS activity by ELISA Kit. The GUS activity (U/g) is determined by comparing the Optical Density of the samples to the standard curve generated from standard with kit. Percentage % = No. of shoots in each class/total number × 100%. ^a^ Total GUS-positive shoots means the shoots with GUS staining, including +, ++ and +++. (–) represents no L-amino acid added into the media. (+) represents L-amino acid added into the media.

**Table 3:** No. of elongated shoots at different times and elongation rates in the different treatments.

Treatment	No. of Explants	No. of Elongated Plants (Percentage, %)						Elongation Rate %
		Total	SE1	SE2	SE3	SE4	SE5	
(−) no supplemental l-amino acid	390	74	21 (28.4)	28 (37.8)	25 (33.8)	-	-	20.2 ± 5.6 ^b^
(+) l-asparagine	407	162	56 (34.6)	71 (43.8)	31 (19.1)	4 (2.5)	-	39.8 ± 1.9 ^ab^
(+) l-glutamine	358	116	16 (13.8)	43 (37.1)	40 (34.5)	13 (11.2)	4 (3.4)	26.6 ± 11.1 ^ab^
(+) l-asparagine and l-glutamine	431	180	24 (13.3)	104 (57.7)	35 (19.5)	17 (9.5)	-	41.6 ± 18.2 ^a^

Three independent experiments were performed for each treatment. No. of explants: the total number of explants for the three experiments; No. of elongated plants: the total number of elongated plants for the three experiments. SE1: the first time; SE2: the second time; SE3: the third time; SE4: the fourth time; SE5: the fifth time. Percentage % = No. of elongated shoots produced at a specific time/Total number ×100%. The elongation rate is expressed as the mean ± standard deviation. The different small letters represent significant differences between treatments by ANOVA (*p* < 0.05). (–) represents no l -amino acid added into the media. (+) represents L-amino acid added into the media.

**Table 4:** Detection of elongated plants and transformation frequency for the different treatments.

Treatment	No. of Explants	No. of Elongated Plants	No. of Positive Plants	Positive Rate of Elongated Plants (%)	Transformation Efficiency (%)
(−) no supplemental l-amino acid	390	74	12	16.2 ± 6.8 ^b^	3.5 ± 2.4 ^c^
(+) l-asparagine	407	162	22	14.8 ± 4.8 ^b^	5.9 ± 2.1 ^b,c^
(+) l-glutamine	358	116	32	36.0 ± 11.6 ^a^	8.8 ± 1.5 ^a,b^
(+) l-asparagine and l-glutamine	431	180	46	30.2 ± 14.9 ^a,b^	11.1 ± 2.7 ^a^

Each treatment has three independent experiments. No. of infected explants: the total number of infected explants for the three experiments; No. of elongated explants: the total number of elongated explants for the three experiments. No. of positive plants: the total number of plants with PAT strips^+^. Positive rate of elongated plants % = No. of positive plants/No. of elongated explants × 100%. The transformation frequency % = the No. of positive plants/No. of infected explants × 100%. The Positive rate of elongated plants and transformation frequency are expressed as the mean ± standard deviation. The different small letters represent significant differences between treatments by ANOVA (*p* < 0.05). (–) represents no L-amino acid added into the media. (+) represents L-amino acid added into the media. ^+^ represents positive.

**Table 5:** Shoot induction frequency and transformation frequency in four soybean cultivars.

Genotype	Shoot Induction Frequency (%)		Transformation Frequency (%)	
	(−) No Supplemental l-Amino Acid	(+) l-Asparagine and l-Glutamine	(−) No Supplemental l-Amino Acid	(+) l-Asparagine and l-Glutamine
Jack	81.7 ± 3.8 ^b^	92.3 ± 1.2 ^a^	5.5 ± 0.6 ^b^	11.1 ± 2.9 ^a^
Zigongdongdou	56.3 ± 4.3 ^a^	62.7 ± 3.4 ^a^	1.3 ± 0.02 ^a^	2.6 ± 1.3 ^a^
Williams82	81.6 ± 1.5 ^b^	86.8 ± 1.5 ^a^	4.4 ± 0.7 ^b^	8.3 ± 1.9 ^a^
Heihe27	79.0 ± 2.0 ^b^	85.7 ± 2.6 ^a^	4.9 ± 1.2 ^a^	8.2 ± 1.8 ^a^

Three independent experiments were performed for each genotype. The shoot induction frequency and transformation frequency were expressed as the mean ± standard deviation. The different small letters represent significant differences between treatments by ANOVA (*p* < 0.05). (–) represents no L-amino acid added into the media. (+) represents L-amino acid added into the media.

**Table 6:** Segregation of *bar* gene in the T1 plants for the different treatments.

Treatment	T0 Lines	No. of T1 Plants	Segregation Pattern				
			*bar^+^*	*bar* *^–^*	Ratio	χ^2^	*p*-Value
(−) no supplemental l-amino acid	1	40	29	11	3:1	0.1333	0.7150
	2	12	11	1	-	-	-
	3	11	9	2	-	-	-
	4	40	0	40	-	-	-
	5	13	0	13	-	-	-
(+) l-asparagine	6	50	13	37	-	-	-
	7	36	28	8	3:1	0.1481	0.7003
	8	45	42	3	-	-	-
	9	30	0	30	-	-	-
	10	50	2	48	-	-	-
(+) l-glutamine	11	17	15	2	-	-	-
	12	14	13	1	-	-	-
	13	40	13	27	-	-	-
	14	35	1	34	-	-	-
	15	25	0	25	-	-	-
(+) l-asparagine and l-glutamine	16	13	10	3	-	-	-
	17	40	31	9	3:1	0.1333	0.7150
	18	10	1	9	-	-	-
	19	20	1	19	-	-	-
	20	15	0	15	-	-	-

Each T0 line represents an independent transformant. T0 lines (1–5) from treatment without l-asparagine and l-glutamine; T0 lines (6–10) from treatment with l-asparagine; T0 lines (11–15) from treatment with l-glutamine; T0 lines (16–20) from treatment with both l-asparagine and l-glutamine. No. of T1 plants: the number of seeds from each T0 line. Three lines (line 1, line 7, line 17) showed a 3:1 ratio (*bar^+^*: *bar^−^*).Lines which were not fit to this ratio were represented by “-”. (–) represents no L-amino acid added into the media. (+) represents L-amino acid added into the media.

**Table 7:** Inheritance frequency of T0 transformants for the different treatments.

Treatment	No. of T0 Transformants	No. of Inheritance Transformants	Inheritance Frequency (%)
(−) no supplemental l-amino acid	12	8	66.7 ± 25.5 ^a^
(+)l-asparagine	22	15	68.2 ± 6.3 ^a^
(+) l-glutamine	32	23	71.9 ± 15.8 ^a^
(+) l-asparagine and l-glutamine	46	34	73.9 ± 5.5 ^a^

Three independent experiments were performed for each genotype. The inheritance frequency was expressed as the mean ± standard deviation. The different small letters represent significant differences between treatments by ANOVA (*p* < 0.05). (–) represents no L-amino acid added into the media. (+) represents L-amino acid added into the media.

## Abbreviations

GCM	germination culture medium
LCCM	liquid co-cultivation medium
CCM	co-culture medium
SIM0	recovery medium
SIM6	selection culture medium
SEM	shoot elongation medium
RCM	rooting culture medium
MES	2-(*N*-morpholino)ethanesulfonic acid
DTT	dl-dithiothreitol
AS	3′,5′-dimethoxy-4-hydroxyacetophenone
6-BA	6-benzylaminopurine
GA	gibberellic acid
IAA	3-indoleacetic acid
IBA	indole-3-butytric acid

## References

[1] Homrich M.S., Wiebke-Strohm B., Weber R.L., Bodanese-Zanettini M.H. (2012). Soybean genetic transformation: A valuable tool for the functional study of genes and the production of agronomically improved plants. Genet. Mol. Biol..

[2] James C. (2015). 20th Anniversary (1996 to 2015) of the Global Commercialization of Biotech Crops and Biotech Crop Highlights in 2015.

[3] Xia Z., Zhai H., Lü S., Wu H., Zhang Y. (2013). Recent achievement in gene cloning and functional genomics in soybean. Sci. World J..

[4] Shan Q., Wang Y., Li J., Zhang Y., Chen K., Liang Z., Zhang K., Liu J., Xi J.J., Qiu J.L. (2013). Targeted genome modification of crop plants using a CRISPR-Cas system. Nat. Biotechnol..

[5] Jiang W., Zhou H., Bi H., Fromm M., Yang B., Weeks D.P. (2013). Demonstration of CRISPR/Cas9/sgRNA-mediated targeted gene modification in *Arabidopsis*, tobacco, sorghum and rice. Nucleic Acids Res..

[6] Feng Z., Zhang B., Ding W., Liu X., Yang D.L., Wei P., Cao F., Zhu S., Zhang F., Mao Y. (2013). Efficient genome editing in plants using a CRISPR/Cas system. Cell Res..

[7] Miao J., Guo D., Zhang J., Huang Q., Qin G., Zhang X., Wan J., Gu H., Qu L.J. (2013). Targeted mutagenesis in rice using CRISPR-Cas system. Cell Res..

[8] Xie K., Zhang J., Yang Y. (2014). Genome-wide prediction of highly specific guide RNA spacers for CRISPR-Cas9-mediated genome editing in model plants and major crops. Mol. Plant.

[9] Upadhyay S.K., Kumar J., Alok A., Tuli R. (2013). RNA-guided genome editing for target gene mutations in wheat. G3.

[10] Wang Y., Cheng X., Shan Q., Zhang Y., Liu J., Gao C., Qiu J.L. (2014). Simultaneous editing of three homoeoalleles in hexaploid bread wheat confers heritable resistance to powdery mildew. Nat. Biotechnol..

[11] Liang Z., Zhang K., Chen K., Gao C. (2014). Targeted mutagenesis in *Zea mays* using TALENs and the CRISPR/Cas system. J. Genet. Genom..

[12] Svitashev S., Young J.K., Schwartz C., Gao H., Falco S.C., Cigan A.M. (2015). Targeted mutagenesis, precise gene editing, and site-specific gene Insertion in maize using Cas9 and guide RNA. Plant Physiol..

[13] Jacobs T.B., LaFayette P.R., Schmitz R.J., Parrott W.A. (2015). Targeted genome modifications in soybean with CRISPR/Cas9. BMC Biotechnol..

[14] Sun X., Hu Z., Chen R., Jiang Q., Song G., Zhang H., Xi Y. (2015). Targeted mutagenesis in soybean using the CRISPR-Cas9 system. Sci. Rep..

[15] Cai Y., Chen L., Liu X., Sun S., Wu C., Jiang B., Han T., Hou W. (2015). CRISPR/Cas9-mediated genome editing in soybean hairy roots. PLoS ONE.

[16] Li Z., Liu Z.B., Xing A., Moon B.P., Koellhoffer J.P., Huang L., Ward R.T., Clifton E., Falco S.C., Cigan A.M. (2015). Cas9-Guide RNA Directed Genome Editing in Soybean. Plant Physiol..

[17] Michno J.M., Wang X., Liu J., Curtin S.J., Kono T.J., Stupar R.M. (2015). CRISPR/Cas mutagenesis of soybean and *Medicagotruncatula* using a new web-tool and a modified Cas9 enzyme. GM Crops Food.

[18] Tang F., Yang S., Liu J., Zhu H. (2016). Rj4, a gene controlling nodulation specificity in soybeans, encodes a thaumatin-like protein but not the one previously reported. Plant Physiol..

[19] Du H., Zeng X., Zhao M., Cui X., Wang Q., Yang H., Cheng H., Yu D. (2016). Efficient targeted mutagenesis in soybean by TALENs and CRISPR/Cas9. J. Biotechnol..

[20] Hinchee M.A., Connor-Ward D.V., Newell C.A., McDonell R.E., Sato S.J., Gasser C.S., Fishhoff D.A., Re D.B., Fraley R.T., Horsch R.B. (1988). Production of transgenic soybean plants using *Agrobacterium*-mediated DNA transfer. Nat. Biotechnol..

[21] McCabe D.E., Swain W.F., Martinell B.J., Christou P. (1988). Stable transformation of soybean (*Glycine max*) by particle acceleration. Nat. Biotechnol..

[22] Shri M., Rai A., Verma P.K., Misra P., Dubey S., Kumar S., Verma S., Gautam N., Tripathi R.D., Trivedi P.K. (2013). An improved *Agrobacterium*-mediated transformation of recalcitrant indica rice (*Oryza sativa* L.) cultivars. Protoplasma.

[23] Cheng M., Lowe B.A., Spencer T.M., Ye X.D., Armstrong C.L. (2004). Factors influencing Agrobacterium-mediated transformation of monocotyledonous species. In Vitro Cell. Dev. Biol-Plant.

[24] Paz M.M., Martinez J.C., Kalvig A.B., Fonger T.M., Wang K. (2006). Improved cotyledonary node method using an alternative explants derived from mature seed for efficient *Agrobacterium*-mediated soybean transformation. Plant Cell Rep..

[25] Wang G., Xu Y. (2008). Hypocotyl-based *Agrobacterium*-mediated transformation of soybean (*Glycine max*) and application for RNA interference. Plant Cell Rep..

[26] Liu H.K., Yang C., Wei Z.M. (2004). Efficient *Agrobacterium tumefaciens*-mediated transformation of soybeans using an embryonic tip regeneration system. Planta.

[27] Meurer C.A., Dinkins R.D., Collins G.B. (1998). Factors affecting soybean cotyledonary node transformation. Plant Cell Rep..

[28] Zhang Z., Xing A., Staswick P.E., Clemente T.E. (1999). The use of glufosinate as a selective agent in *Agrobacterium*-mediated transformation of soybean. Plant Cell Tissue Organ. Cult..

[29] Clemente T.E., LaVallee B.J., Howe A.R., Conner-Ward D., Rozman R.J., Hunter P.E., Broyles D.L., Kasten D.S., Hinchee M.A. (2000). Progeny analysis of glyphosate selected transgenic soybean derived from Agrabacterium-mediated transformation. Crop Sci..

[30] Olhoft P.M., Flagel L.E., Donovan C.M., Somers D.A. (2003). Efficient soybean transformation using hygromycin B selection in the cotyledonary-node method. Planta.

[31] Liu S.J., Wei Z.M., Huang J.Q. (2008). The effect of co-cultivation and selection parameters on *Agrobacterium*-mediated transformation of Chinese soybean varieties. Plant Cell Rep..

[32] Arun M., Subramanyam K., Mariashibu T.S., Theboral J., Shivanandhan G., Manickavasagam M., Ganapathi A. (2015). Application of sonication in combination with vacuum infiltration enhances the *Agrobacterium*-mediated genetic transformation in India soybean cultivars. Appl. Biochem. Biotechnol..

[33] Zeng P., Vadnais D.A., Zhang Z., Polacco J.C. (2004). Refined glufosinate selection in *Agrobacterium*-mediated transformation of soybean [*Glycine max* (L.) Merrill]. Plant Cell Rep..

[34] Zhang W.J., Dewey R.E., Boss W., Phillippy B.Q., Qu R. (2013). Enhanced *Agrobacterium*-mediated transformation efficiencies in monocot cells is associated with attenuated defense responses. Plant Mol. Biol..

[35] Wu C.C., Singh P., Chen M.C., Zimmerli L. (2010). L-Glutamine inhibits beta-aminobutyric acid-induced stress resistance and priming in *Arabidopsis*. J. Exp. Bot..

[36] Pageau K., Reisdorf-Cren M., Morot-Gaudry J.F., Masclaux-Daubresse C. (2006). The two senescence-related markers, GSI and GDH, involved in nitrogen mobilization, are differentially regulated during pathogen attack and by stress hormones and reactive oxygen species in *Nicotianatabacum* L. leaves. J. Exp. Bot..

[37] Zhang J., Fan J., Venneti S., Cross J.R., Takagi T., Bhinder B., Djaballah H., Kanai M., Cheng E.H., Judkins A.R. (2014). Asparagine plays a critical role in regulating cellular adaptation to glutamine depletion. Mol. Cell.

[38] Graham M.Y., Weidner J., Wheeler K., Pelow M.J., Graham T.L. (2003). Induced expression of pathogenesis-related protein genes in soybean by wounding and the *Phytophthorasojae* cell wall glucan elicitor. Physiol. Mol. Plant P..

[39] Graham M.Y. (2005). The diphenylether herbicide lactofen induces cell death and expression of defense-related genes in soybean. Plant Physiol..

[40] Møller M.G., Taylor C., Rasmussen S.K., Holm P.B. (2003). Molecular cloning and characterization of two genes encoding asparagine synthetase in barly (*Hordeumvulgare* L.). Biochim. Biophys. Acta.

[41] Lam H.M., Coschigano K., Schultz C., Melo-Oliveria R., Tjaden G., Oliveira I., Ngai N., Hsieh M.H., Coruzzi G. (1995). Use of *Arabidopsis* mutants and genes to study amide amino acid biosynthesis. Plant Cell.

[42] Lam H.M., Peng S.S., Coruzzi G.M. (1994). Metabolic regulation of the gene encoding glutamine-dependent asparagine synthetase in *Arabidopsis thaliana*. Plant Physiol..

[43] Liu G., Ji Y., Bhuiyan N.H., Pilot G., Selvaraj G., Zou J., Wei Y. (2010). Amino acid homeostasis modulates salicylic acid-associated redox status and defense responses in *Arabidopsis*. Plant Cell.

[44] Song Z.Y., Tian J.L., Fu W.Z., Li L., Lu L.H., Zhou L., Shan Z.H., Tang G.X., Shou H.X. (2013). Screening Chinese soybean genotypes for *Agrobacterium*-mediated genetic transformation suitability. J. ZhejiangUniv. Sci. B..

[45] Cai Y., Chen L., Liu X., Guo C., Sun S., Wu C., Jiang B., Han T., Hou W. (2018). CRISPR/Cas9-mediated targeted mutagenesis of *GmFT2a* delays flowering time in soya bean. Plant Biotechnol. J..

[46] Di R., Purcell V., Collins G.B., Ghabril S.A. (1996). Production of transgenic soybean lines expressing the bean pod mottle virus coat protein precursor gene. Plant Cell Rep..

[47] Frame B.R., Shou H.X., Chikwamba R.K., Zhang Z., Xiang C., Fonger T.M., Pegg S.E.K., Li B., Nettleton D.S., Pei D. (2002). *Agrobacterium tumefaciens*-mediated transformation of maize embryos using standard binary vector system. Plant Physiol..

[48] Jefferson R.A. (1987). Assaying chimeric genes in plants: The *GUS* gene fusion system. Plant Mol. Biol. Rep..

